# Toxins and virulence factors of enterotoxigenic *Escherichia coli* associated with strains isolated from indigenous children and international visitors to a rural community in Guatemala

**DOI:** 10.1017/S0950268814002295

**Published:** 2014-09-19

**Authors:** O. R. TORRES, W. GONZÁLEZ, O. LEMUS, R. A. PRATDESABA, J. A. MATUTE, G. WIKLUND, D. A. SACK, A. L. BOURGEOIS, A-M. SVENNERHOLM

**Affiliations:** 1 Institute for Nutrition in Central America and Panama (INCAP), Guatemala City, Guatemala; 2 Universidad de San Carlos de Guatemala, Facultad de Ciencias Quimicas y Farmacia, Escuela de Quimica Biologica, Guatemala City, Guatemala; 3 Centro de Investigaciones en Nutrición y Salud (CIENSA), Guatemala City, Guatemala; 4 Department of Microbiology and Immunology, University of Gothenburg, Gothenburg, Sweden; 5 Johns Hopkins Bloomberg School of Public Health, Baltimore, MD, USA

**Keywords:** Diarrhoea, enteric bacteria, enterotoxin, *Escherichia coli*, travellers' infection

## Abstract

Diarrhoea remains a common cause of illness in Guatemala, with children suffering most frequently from the disease. This study directly compared the frequency, enterotoxin, and colonization factor (CF) profiles of enterotoxigenic *Escherichia coli* (ETEC) strains isolated from children living in a rural community in Guatemala and from Western visitors to the same location during the same seasons, using similar detection methodologies. We found that ETEC accounted for 26% of severe cases of diarrhoea in children requiring hospitalization, 15% of diarrhoea in the community, and 29% of travellers' diarrhoea in visitors staying ⩾2 weeks. The toxin and CF patterns of the ETEC strains isolated from both groups differed significantly (*P* < 0·0005) as determined by *χ*^2^ = 60·39 for CFs and *χ*^2^ = 35 for toxins, while ETEC phenotypes found in Guatemalan children were comparable to those found in children from other areas of the world.

## INTRODUCTION

Guatemala is a developing country located close to the USA, with great natural beauty and popular tourist destinations to which 1·8 million foreign visitors travel every year. The population of Guatemala is 14·7 million, of which 40–50% are children and adolescents, and the World Bank classifies the country at the lower middle-income level [1]. The 2012 Human Development Index (HDI) value for Guatemala was 0·581, which places the country in the ‘medium’ human development category [2]. (According to the United Nations Development Programme, the HDI is an average measure of basic human development achievements in a country.) Between 1980 and 2012, Guatemala's HDI value experienced an average annual increase of only about 0·9%. As such, the country's overall development progress has been slow in the past few decades, and it remains among the lesser-developed countries found in the same region and development group [2].

Enterotoxigenic *Escherichia coli* (ETEC) has been implicated as the cause of about 400 million cases of diarrhoea and at least 150 000– 300 000 deaths annually in pre-school-aged children of developing countries [3]. ETEC has also been shown to be an important cause of diarrhoeal disease associated with morbidity and mortality in older age groups in developing country settings. In fact, studies in endemic areas suggest that ETEC along with cholera may contribute to about half of the 1·15 million diarrhoea-associated deaths estimated to occur in individuals aged >5 years [3–5]. In endemic areas, like Bangladesh, it has also been reported that ETEC is the most frequently isolated pathogen during the first 2 years of life in children [6]. ETEC is also the most common cause of travellers' diarrhoea (TD) in visitors to ETEC-endemic countries, accounting for 20–50% of all such episodes [3, 4]. Guatemala's children frequently suffer from diarrhoea, which is the country's second-leading cause of child morbidity and mortality, with rates as high as 8–11 episodes/child per year [7].

The burden of ETEC illness and the phenotypic characteristics of the strains associated with illness in indigenous children, as well as in travellers, have often been difficult to assess because of the relative complexity of the laboratory methods required to identify enterotoxin-producing strains and the wide variety of colonization factors (CFs) that exist [4, 8–10]. This study was undertaken to evaluate the importance of ETEC as a diarrhoeal pathogen in Guatemalan children and travellers to Guatemala.

The participating indigenous children were drawn from the rural community of Santa María de Jesús, Sacatepéquez, which is located ~10 km from Antigua. Participating children were also identified from emergency-room patients at Hospital Roosevelt, the major pediatric hospital in Guatemala City, or from patients of the zone 11 clinic of the Instituto Guatemalteco de Seguridad (IGSS), also in Guatemala City. Both of these sites are located ~39 km from Antigua. The participating travellers were adults originally from the USA and Canada, who were visiting Antigua, Guatemala, for Spanish language courses or vacation.

At the time of our data collection (in 2002), Guatemala City had approximately 2·5 million inhabitants and, as the capital of Guatemala, it is considered a modern city. Antigua is the capital of Sacatepéquez, a central highlands department of Guatemala with nearly 250 000 inhabitants. It is a tourist destination with many foreign visitors who are drawn to its ‘old-world’ feel with no buses or tall buildings, although tourism has brought Sacatepéquez internet cafes, hotels with modern amenities, and affluence. Santa María de Jesús is a small village located at the foot of Volcán de Agua, also in the department of Sacatepéquez, with more than 15 000 inhabitants who are mainly Kakchiquel, one of the indigenous Maya peoples of the midwestern highlands in Guatemala, who follow their traditional Indian customs.

Although this data was collected about 10 years ago (November 1999 to October 2003), we believe that the results will be valuable in helping to implement improved diarrhoeal disease control and prevention efforts. The proportion of child deaths in Guatemala attributed to diarrhoea have decreased modestly over the last 10 years (from 11% in 2000 to 7% in 2011) [11]; however, it is still a major cause of morbidity and mortality and requires increased attention. In addition, a recent TD vaccine trial in Guatemala found that ETEC remains a significant cause of diarrhoea in visitors to the country [12–14]. From a more global perspective, recent studies, including whole-genome sequencing of a large number of ETEC at the Sanger Institute, have shown that ETEC lineages have remained stable in different geographical locations over time (A-M. Svennerholm, personal communication) and recent studies from Bangladesh and Egypt show rather similar toxin and CF profiles of clinical ETEC isolates over time [15, 16]. Based on these data, along with the development indicators cited earlier, we assert that there has probably been very little change in the risk of experiencing ETEC diarrhoea in Guatemala, both for children and travellers, since conducting this study. Therefore, the results of this study still present a representative picture of ETEC disease burden in Guatemala and may help to inform the design and evaluation of new ETEC vaccines [17] for infants and young children living in endemic areas, as well as for international visitors to these regions.

## METHODS

We identified severe cases of diarrhoea for 1 year at Hospital Roosevelt in Guatemala City in children aged 6–36 months whose mothers voluntarily signed an informed consent document. We followed community cases by passive surveillance during two consecutive years in the community of Santa María de Jesús (SMJ), Sacatepéquez, in children whose mothers voluntarily signed an informed consent and provided a diarrhoea sample. For community children, non-diarrhoea controls were matched for age, gender, and residence zone with children whose mothers believed they were healthy and that had not suffered from diarrhoea for at least 2 months previously, as well as the mothers' willingness to collect and provide the study personnel with a formed stool sample.

Data from visitors to Antigua, Guatemala, located 10 km away from SMJ, are from two TD vaccine trials and one plurifloxacin trial, conducted by some of the authors of this paper [18–20]. The duration of exposure for the travellers' studies was more short-term in nature, in that subjects were generally on-site for only 14–28 days, as opposed to the children being residents of the area. For the vaccine and antibiotic treatment studies, only ETEC isolates from a subject's initial acute diarrhoea episode in Guatemala were included in the analysis presented here. ETEC isolates classified as coming from asymptomatic subjects were from individuals who did not have a history of diarrhoeal illness while in Guatemala.

ETEC was isolated from the samples in Guatemala City at the Institute for Nutrition in Central America and Panama (INCAP) microbiology laboratory, following standard methods described in previous publications [21, 22]. During the course of these studies, the INCAP laboratory also participated in an externally monitored (by Johns Hopkins Bloomberg School of Public Health) enteric pathogen proficiency testing programme, which was conducted on a monthly basis. For the visitors, toxin types and CF antigens were determined by the GM1 enzyme-linked immunoassay (GM1-ELISA) and the dot-blot assay at the University of Gothenburg in Sweden. For the children, the same methods were implemented at INCAP with careful external and internal quality control between the INCAP and Gothenburg laboratories. Thus, all toxin-positive strains identified in Guatemala were sent to the World Health Organization reference laboratory in Gothenburg for confirmation of toxin profiles by GM1-ELISA (the same methods and reagents were used at INCAP and at Gothenburg). About 10% of the negative samples were also sent to the Gothenburg laboratory for confirmation as an additional quality control check. The CF profiles of the toxin-positive ETEC strains isolated from both travellers and indigenous subjects in Guatemala were confirmed by dot-blot analyses at the Gothenburg laboratory. All monoclonal antibodies used in these studies were originally developed in Gothenburg, and the same batches of antibodies were used there and at INCAP. The experiences of the Gothenburg laboratory have shown good agreement between using the dot-blot method for determining CF profiles and more molecular-based CF detection methods [22–24].

Statistical analyses were conducted to determine if differences existed between the three study groups with regard to the prevalence of heat-labile toxin (LT)-only, heat-stable toxin (ST)-only, and combination LT/ST strains, with or without various CFs. The significance of six combinations, including the odds ratios related to the presence of LT and ST outcome and diarrhoea, were calculated and evaluated with Pearson's *χ*^2^ tests using exact *P* values with StatXact v. 4·02 (Cytel Software Corporation, USA) for visitors *vs.* children from the community and children from the hospital with acute diarrhoea *vs*. children with diarrhoea from the community.

## RESULTS

A pathogen was detected in about 50% of all specimens tested from acutely ill travellers and from 83% to 52% of cases and 49% of controls in the paediatric study groups. As shown in Table 1, ETEC was the most frequently isolated bacterial pathogen in both hospitalized children who had not received antibiotics before culture (identified in 26·5% of specimens) and in community children with acute (16·0% of specimens) or persistent (21·7% of specimens) diarrhoea, as well as a non-diarrhoea control group (10·2% of specimens). ETEC was also the most frequently isolated pathogen in the visitors' group (identified in 29·1% of specimens). *Campylobacter jejuni/coli* were the most common bacterial pathogens isolated from asymptomatic children in SMJ (20·2%), most of whom had frequent contact with domestic livestock and/or poultry living in close proximity to their homes.

**Table 1.** Relative distribution of enteropathogens in Guatemalan children and visitors to Guatemala with diarrhoea

	Children without diarrhoea in SMJ (*n* = 109)	Children with acute diarrhoea in SMJ (*n* = 543)	Children with persistent diarrhoea in SMJ (*n* = 97)	Hospitalized children with severe diarrhoea (*n* = 83)	Adult visitors/travellers with diarrhoea* (*n* = 230)
Enterotoxigenic *Escherichia coli*	11 (10·1%)	87 (16·0%)	26 (21·7%)	20 (26·5%)	67 (29·1%)
*Campylobacter jejuni/coli*	22 (20·2%)	74 (13·6%)	18 (18·6%)	4 (4·8%)	21 (9·1%)
*Salmonella* spp.	2 (1·8%)	1 (0·2%)	2 (2·1%)	1 (1·2%)	11 (4·8%)
*Shigella* spp.	2 (1·8%)	38 (7%)	0	5 (7·2%)	13 (5·6%)
Parasites	16 (14·7)	173 (31·9)†	29 (29·9%)†	13 (15·7%)‡	5 (2·1%)
Rotavirus	n.t.	8 (1·5%)	0	17 (20·5%)	0
Adenovirus	n.t.	n.t.	n.t.	9 (10·8%)	n.t.
No pathogen	56 (51·3%)	236 (43%)	47 (48%)	14 (16·9%)	113(49·1%)

SMJ, Santa María de Jesús; n.t., not tested.

* By active or passive surveillance.

† Main parasites found in SMJ were helminths.

‡ Main parasite found in hospitalized children was *Cryptosporidia*.

Stool samples from 640 cases of diarrhoea and 109 non-diarrhoea controls from SMJ were evaluated. Infection with LT strains, either alone or in combination with ST, in these children was associated with diarrhoea [odds ratio (OR) 2·68, 95% confidence interval (CI) 1·05–8·69, *P* = 0·037]. However, infection with ST-only strains was not associated with acute diarrhoeal disease in community children compared to asymptomatic controls (*P* = 0·3496, non-significant). In the diarrhoea cases, 543 met the criteria for acute diarrhoea while 97 (15·2% of all community cases) met the criteria for persistent diarrhoea (>14 days duration). In the acute cases, LT-only strains (7·6%) were the most common, followed by ST-only strains (4·8%) and LT/ST strains (3·7%), respectively (Table 2). In persistent diarrhoea cases, LT-only strains were again the most common isolates. Mixed infections with LT-only strains accounted for 12 additional cases to those shown in Table 2. The CF distribution of ETEC strains isolated from cases and controls in SMJ was varied and a large percentage of the 116 ETEC strains analysed for CFs lacked a detectable CF (64·8%. Table 3). The most commonly expressed CF on community ETEC strains was CS6, which was identified in 11 (9·5%) of the strains, followed by CS1+CS3 or CS2+CS3 in 6·9% and CS4+CS6 or CS5+CS6 in 6·0% of the strains; other CFs were only found in low frequencies, i.e. in 2–4 of the 116 strains (Table 3).

**Table 2.** Toxin profiles of ETEC isolated from different study groups of Guatemalan children and visitors to Guatemala

	Children without diarrhoea in SMJ (*n* = 109)	Children with acute diarrhoea in SMJ (*n* = 543)	Children with persistent diarrhoea in SMJ (*n* = 97)	Children hospitalized with severe diarrhoea in Guatemala city (*n* = 83)*	Visitors without diarrhoea (vaccine trial, placebo recipents) (*n* = 269	Visitors with diarrhoea (vaccine trial, placebo recipients) (*n* = 126)	Visitors with diarrhoea (antibiotic trial) (*n* = 104)
LT only	4 (3·7%)	41 (7·6%)	12 (12·4%)	8 (9·6%)	4 (1·5%)	6 (4·8%)	3 (3%)
ST only	6 (5·5%)	26 (4·6%)	5 (5·2%)	6 (7·2%)	12 (4·5%)	18 (14·3%)	10 (10%)
LT/ST	1 (0·9%)	20 (3·7%)	4 (4·1%)	8 (9·6%)	1 (0·4%)	7 (5·6%)	10 (10%)
Total	11 (10·1%)	87 (15·3%)	21 (21·7%)	22 (26·5%)	20 (7·4%)†	38 (30·2%)‡	29 (28%)§

ETEC, Enterotoxigenic *Escherichia coli*; SMJ, Santa María de Jesús; LT, heat-labile toxin; ST, heat-stable toxin.

* These children had not received antimicrobials as an inclusion criteria to the study.

† Three additional cases were of mixed toxin phenotype or were mixed with another enteropathogen.

‡ Seven additional cases were of mixed toxin phenotype or were mixed with another enteropathogen.

§ Six additional cases were of mixed toxin phenotype or mixed with another enteropathogens.

**Table 3.** Colonization factors (CFs) on ETEC isolated from children in Santa María de Jesús

		CFA/I	CS1, CS2, CS3	CS4, CS5, CS6	CS17	CS12	CS14	CS6	CS7	CS17	No CF	Total
Persistent diarrhoea	LT only	0	0	0	0	0	0	0	1	0	11	12
	ST only	0	1	1	0	0	0	2	0	0	1	5
	LT/ST	0	2	0	0	0	0	1	0	0	1	4
	Total %	0	3	1	0	0	0	3	1	0	13	21
Diarrhoea	LT only	0	0	0	4	0	0	0	0	1	38	43
	ST only	1	0	3	0	0	2	6	0	0	11	26
	LT/ST	2	4	0	0	2	0	0	0	0	9	17
	Total %	3	4	3	4	2	2	6	0	1	58	86
Non-diarrhoea controls	LT only	0	1	0	0	0	0	1	0	0	2	2
	ST only	0	0	0	0	0	1	2	0	0	3	5
	LT/ST	1	0	0	0	0	0	0	0	0	1	2
	Total %	1	1	0	0	0	1	3	0	0	3	9

ETEC, Enterotoxigenic *Escherichia coli*; LT, heat-labile toxin; ST, heat-stable toxin.

Of the ETEC strains isolated from the hospitalized children, the most prevalent toxin profiles were LT-only and LT/ST, each accounting for 9·6% of the diarrhoea cases; ST-only strains were recovered from 7·2% of the cases (Table 2). Compared to asymptomatic controls, LT and LT/ST strains were again very strongly associated with severe illness in the hospitalized study group (OR 4·97, 95% CI 1·62–18·02, *P* = 0·026). Mixed infections were found in 6/20 of the ETEC hospital cases. Of the ETEC strains isolated from these children, CFA/I was the most common CF, found in 7/22 (31·8%) of the strains, followed by CS1–3 in 2/22 (9%) of the strains and CS14 and CS17 in 1/22 strains each (both 4·5%) (Table 4). Nine (41%) of 22 of the ETEC strains isolated from the hospitalized children had no detectable CF (Table 4).

**Table 4.** Colonization factors (CFs) on ETEC isolated from hospitalized children in Guatemala

	CFA/I	CS1, CS2, CS3	CS14	CS17	No CF	Frequency of CF-positive ETEC
LT only	0	0	0	0	8 (100%)	0/8
ST only	3 (50%)	0	1 (16.7%)	0	2 (33.3%)	4/6
LT/ST	4 (50%)	2 (25%)	0	1 (12.5%)	1 (12.5%)	7/8
Total	7 (32%)	2 (9%)	1 (4.5%)	1 (4.5%)	9 (41%)	11/22 (50%)

ETEC, Enterotoxigenic *Escherichia coli*; LT, heat-labile toxin; ST, heat-stable toxin.

Sixty-seven ETEC strains were isolated from visitors meeting the TD case definition and from 20 subjects with asymptomatic infection acquired while visiting Antigua, Guatemala (Table 2). Given the travellers' relatively short duration of exposure to diarrhoea-causing pathogens, multiple diarrhoea episodes were quite rare. Of the 87 ETEC isolates from the visitors, 38 were from TD cases identified by active surveillance methods in placebo recipients participating in two different vaccine trials [19, 20], 29 were from TD cases identified by passive surveillance in an antibiotic treatment study [21], and 20 were recovered from asymptomatic travellers participating in the vaccine trial [19, 20]. ETEC infection was significantly more prevalent in visitors to Guatemala with diarrhoea than in non-diarrhoea controls; this was true for all toxin combinations: LT/ST (OR 21·39, 95% CI 3·29–896·9, *P* < 0·00001); ST-only (OR 43·99, 95% CI 11·31–374·67, *P* < 0·00001); and LT-only (OR 6·73, 95% CI 2·48–22·76, *P* < 0·00001).

The most common toxin phenotype recovered from TD cases or asymptomatic subjects was ST-only, found in 45/87 isolates (51·7%); 24 (53·3%) of these ST-only strains expressed CS6, three (6·7%) expressed CFA/I, and 18 (40·0%) were devoid of detectable CFs (Table 5). LT-only strains were found in 17/87 (19·5%) of the visitors; one strain expressed CS17 and two expressed CS12, whereas 14 (82·4%) were devoid of detectable CFs. Strains producing both LT and ST accounted for 12/87 (13·8%) of the ETEC isolates; 10 (83·3%) of these expressed either CS3 alone or in combination with CS1 or CS2, one expressed CFA/I (8·3%), and one was devoid of CFs (Table 5). The CF distribution among ETEC strains recovered from asymptomatic visitors mirrored the distribution for the TD cases (data not shown).

**Table 5.** Colonization factors (CFs) on ETEC strains isolated from visitors to Guatemala

	CFA/I	CS1, CS2, CS3	CS4, CS5, CS6	CS17	CS12	CS6	No CF	Mixed	Total
LT only	0	0	0	1	2	0	14	0	17
ST only	3	0	0	0	0	24	18	0	45
LT/ST	1	10	0	0	0	0	1	0	12
Total	4 (4·6%)	10 (11·5%)	0	1 (1·1%)	2 (2·3%)	24 (27·6%)	33 (37·9%)	13 (14·9%)	87 (100%)

ETEC, Enterotoxigenic *Escherichia coli*; LT, heat-labile toxin; ST, heat-stable toxin.

During the surveillance period, ST CS6 strains predominated in the visitor population, while a broader array of toxin and CF profiles was found in the strains isolated from children. Thus, whereas ST-only strains predominated in the visitors (45/87; 52%), about half (53%) of which expressed CS6 only (Tables 2 and 5), LT-only strains were the most commonly isolated in community children (57/116; 49%) followed by ST-only strains (36/116) (Table 2). In the ST-only strains isolated from children, only 10/36 strains (28%) expressed CS6 alone (Table 3). In the hospitalized children, no ST CS6 strains were found (Table 4).

**Fig. 1.**
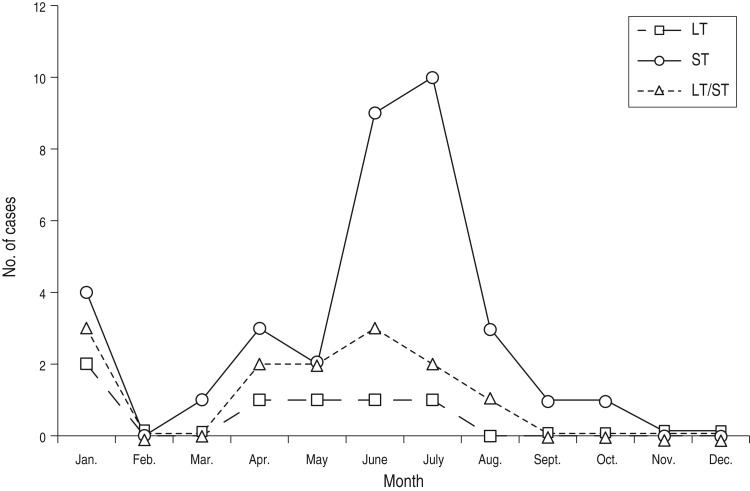
Seasonality of enterotoxigenic *Escherichia coli* infections in children in the community of Santa María de Jesús from June 2001 to October 2003. The pattern shown is representative of the 2-year period. LT, Heat-labile toxin; ST, heat-stable toxin.

Although ETEC strains could be recovered from indigenous children and travellers year-round, a strong seasonal pattern was evident in both the paediatric and visitor study groups with the highest numbers of cases being detected during the rainy months of May to August in both groups. The seasonal pattern for ETEC toxin phenotypes in SMJ is shown in Figure 1. ST-only ETEC predominated during the rainy season in indigenous children. By contrast, LT/ST and LT-producing ETEC were the predominant phenotypes in visitors during the rainy season (data not shown).

## DISCUSSION

This unique study represents the first attempt to directly compare ETEC strains isolated from indigenous children in a highly ETEC-endemic area and from visitors to the same region with regard to toxin and CF profile, during the same seasons and using similar detection methodologies. Our results, which are based on about 3 years of surveillance in both study populations, show that ETEC is a very common pathogen in both children with diarrhoea in Guatemala as well as in international visitors to similar locations in the country. In addition, we observed that, despite the origin of the strains, the pathogens infecting children bear different virulence factors than the strains infecting adult visitors. Moreover, although most ETEC infections and cases in visitors and indigenous children occurred during the rainy season (May–August, see Fig. 1), the predominant toxin phenotype during the peak season differed between the two groups.

The frequency of ETEC infections in children was particularly high in hospitalized children (26·5%), even higher than for rotavirus infections (20%), followed by a high frequency of ETEC in children with persistent diarrhoea (21·7%) and a somewhat lower frequency in community children (15·3%) (Table 1). In two different cohorts of visitors to Guatemala, very similar high frequencies of ETEC infections were also found, with 28% and 30%, respectively of all TD cases associated with ETEC. Interestingly, the frequency of all ETEC and the relative distribution of toxin profiles in the two visitor cohorts were comparable in spite of the fact that these studies were conducted during different years and that ETEC was identified by active and passive surveillance, respectively. When comparing asymptomatic ETEC infections in community children and travellers, comparable frequencies (10·2% and 7·4%, respectively) were also observed (Table 2).

Regarding the asymptomatic subjects, in both the children and visitor study sets the ST-only phenotype predominated and LT/ST infections were rare in both groups (0·9% and 0·4%, respectively). By contrast, in children with diarrhoea from the community of SMJ, LT-only strains were the main ETEC toxin found. Such strains were significantly more common in children than in adult visitors to Guatemala (OR 2·35, *P* = 0·0203). Conversely, visitors were significantly more likely to have diarrhoea due to ST-only infection compared to Guatemalan children (OR 2·27, *P* = 0·0001). ST-only infection, regardless of illness outcome, was also significantly more prevalent in visitors compared to the child populations (OR 2·90, *P* = 0·0001); indeed, adult visitors to Antigua were three times more likely to have a ST-only ETEC infection compared to Guatemalan children. Surprisingly, ST-only infection was not significantly associated with disease in Guatemalan children, regardless of age. However, children aged 0–5 months were not included in the study because they are universally breastfed in SMJ, so we may have missed some illness associated with ST-only strains in this age group. In previous work, we have also documented that both ST_p_- and ST_h_-producing strains are prevalent in Guatemala and both have been recovered from local travellers and indigenous children [25–29]. Moreover, studies conducted with these strains demonstrate that ST CS6 high prevalence is not associated with the presence of a single clone [30]. In studies from other parts of the world, investigators have proposed that ST_p_ may be somewhat less virulent than ST_h_ strains [31]. Based on this information, it could be speculated that many of the ST strains infecting children may have been of the ST_p_ genotype and may have been less able to cause disease. Consequently, this would have made it more difficult to show an association with illness in children in this study.

An interesting observation in this study was the higher frequency of LT-only infections in indigenous children with diarrhoea compared to asymptomatic controls. Other studies in ETEC-endemic areas have not reported a difference in frequencies of LT-only in cases and controls [32, 33]. This apparent difference in the disease association across field sites does not imply that LT-only strains cannot be pathogenic. For example, there have been several reports on the association of LT-only ETEC and diarrhoea during recent years [16, 34] and LT-CS17 has been clearly pathogenic in human challenge studies [35]. Perhaps the inoculum and genetic differences can explain differences in susceptibility.

In spite of the comparable high frequencies of ETEC infections in visitors to Guatemala with TD and indigenous children with diarrhoea of varying severity, the toxin and CF profiles of clinical ETEC isolates differed markedly between the different study groups. Thus, whereas ST-only CS6 strains predominated in the visitor cohorts (27·6% of all ETEC), this phenotype only represented 9·5% of the ETEC strains isolated from community children from SMJ and was not found in the ETEC strains isolated from the hospitalized children. Similarly, a large difference in the prevalence of ST-only strains in travellers and non-travellers in certain geographical areas, as well as a considerably higher proportion of CS6-only ETEC in travellers than in endemic populations, was recently reported in an extensive review on ETEC epidemiology [17]. For the first time in Guatemala, ETEC isolates were characterized in terms of both the toxin type and CF expressed, using comparable laboratory methods implemented and validated locally. These analyses revealed that in children with severe diarrhoea, LT-only and LT/ST strains were the most frequently found phenotypes and that six CFs, i.e. CFA/I, CS1, CS2, CS3, CS17, and CS14, were associated with these highly virulent strains. Thus, severe illness resulting in hospitalization of children in Guatemala was associated with ETEC strains carrying classical CFs similar to those described in other populations of the world [6, 25–30, 36–40].

Among the visitors to Guatemala, a different ETEC toxin and CF distribution than that observed in the children was seen, irrespective of whether active or passive surveillance techniques were used to screen these visitors. ST-only and LT/ST strains were more common than LT-only strains in the visitors, with ST-only CS6 strains being the most common ETEC phenotype.

Knowledge about the prevalence and relative distribution of ETEC and the main virulence factors, i.e. the toxins and CFs, in different populations and geographical areas is of key importance in planning vaccination strategies against ETEC [17, 41]. Based on the results from this study, we can estimate the vaccine CF/CS coverage potentially needed to provide protection against pathogenic ETEC in children in Guatemala as well as among travellers to this ETEC-endemic area. Despite the differences noted above regarding the toxin and CF phenotypes of ETEC strains from the indigenous children and visitors to the same area, it is encouraging that ETEC vaccines formulated to induce immunity to LT toxin and CFA/I, CS3, and CS6 [17, 41] could potentially be useful as a disease-preventive measure in both at-risk groups in Guatemala.

It should be noted that this study has some possible limitations, e.g. the comparatively long time period since samples were collected from the different study groups and that the study was limited to a restricted geographical area. As noted earlier, it has been about 10 years since the samples were collected from the different study groups. However, diarrhoea clearly remains a significant health threat in Guatemala, particularly for indigenous children as well as visitors to the country [11–14]. In addition, the toxin and CF profiles of the ETEC strains collected from travellers participating in three different trials (two different vaccine studies and one antibiotic treatment study) during a prolonged time period were surprisingly similar. This supports the finding that virulence profiles of ETEC isolated from travellers were considerably different from those observed for ETEC isolated from children during the study period in a similar area as well as in numerous other epidemiological studies [17]. In recent studies we have also shown, based on whole genome sequencing of a large number of ETEC strains collected worldwide across three decades, that ETEC lineages are very stable over time and that similar ETEC lineages can be found in Latin America, Asia, and Africa (A. Von Mentzer, unpublished data). Another possible limitation is that the children's study was case-controlled and the visitors' study was prospective; however, we aimed to address this by conducting the study over the same extended time period, in the same geographical locations, and using the same detection methods.

We conclude that ETEC is an important pathogen in Guatemalan children and visitors to Guatemala suffering from diarrhoea. Based on current disease burden and development indicators for Guatemala, as well as more recent TD studies in the country, we believe that there has been very little change in the risk of experiencing ETEC diarrhoea in Guatemala, both for children and travellers, since conducting this study. Therefore, the results still present a representative picture of ETEC disease burden in Guatemala and will be valuable in helping to implement improved diarrhoeal disease control and prevention efforts. The CFA/I ST-only or LT/ST strains were the most predominant ETEC phenotypes (32%) identified in hospitalized children. ETEC strains expressing CFA/I, which is one of the most prevalent ETEC phenotypes [4, 6, 9], were rarely isolated either from community children or visitors. This is surprising since we have previously shown that CFA/I-positive strains were the most common phenotype isolated from children in the neighbouring countries of Nicaragua [26], Peru [27], and Bolivia [28]; however, CS1–CS3- and CS4–CS6-positive strains were the most predominant ETEC phenotypes in a study in travellers to Mexico [18]. The combination of data from this study and these other studies from across Latin America point to the critical importance of ETEC's contribution to diarrhoeal disease burden in the region. Additional studies on ETEC impact and strain identification in this region would help to build the evidence on the associated disease burden and health impacts, so that suitable disease prevention and control strategies, including vaccines, can be rapidly developed and deployed.
